# The Medical News Visiting List for 1894

**Published:** 1894-01

**Authors:** 


THE MEDICAL NEWS VISITING LIST for 1894. Weekly, dated for 30 patients) ; Monthly (undated, for 120 patients per month); Perpetual (undated, for 30 patients weekly per year); and Perpetual (undated, for 60 patients weekly per year). The first three styles contain 32 pages of data and 176 pages of blanks. The 60-Patient Perpetual consists ol 256 pages of blanks. Each style in one wallet-shaped book, pocket, pencil, rubber, and catheter-scale, etc. Seal Grain Leather, $1.25. Philadelphia ; Lea Brothers & Co., 1893.

THE MEDICAL NEWS VISITING LIST for 1894 has been thoroughly revised and brought up to date in every respect. The text portion (32 pages) contains the most useful data for the physician and surgeon, including an alphabetical Table of Diseases, with the most approved Remedies, and a Table of Doses. It also contains sections on Examination of Urine, Artificial Respiration, Incompatibles, Poisons and Antidotes, Diagnostic Table of Eruptive Fevers and the Ligation of Arteries. The classified blanks (176 pages) are arranged to hold records of all kinds of professional work, with memoranda and accounts. Four styles are now published. Weekly (dated, for 30 patients); Monthly (undated, for 120 patients per month, and good for any year) ; Perpetual (undated, for 30 patients weekly per year); and Perpetual (undated, for 60 patients weekly per year.) This last style consists of 256 pages of assorted record blanks, without text. THE MEDICAL NEWS VISITING LIST adapts itself to any system of keeping professional accounts. Each style is in one volume, bound in handsome red leather, with pocket, pencil, rubber, and catheter-scale, price, $1.25. When desired, a Ready Reference Thumb-letter Index is furnished, which is peculiar to this Visiting List, and will save many-fold its small cost (25 cents) in the economy of time effected during a year. In short, every need of the physician seems to have been anticipated in THE MEDICAL NEWS VISITING LIST.

